# Development of In Situ Self-Assembly Nanoparticles to Encapsulate Lopinavir and Ritonavir for Long-Acting Subcutaneous Injection

**DOI:** 10.3390/pharmaceutics13060904

**Published:** 2021-06-18

**Authors:** Irin Tanaudommongkon, Asama Tanaudommongkon, Xiaowei Dong

**Affiliations:** Department of Pharmaceutical Sciences, University of North Texas Health Science Center, Fort Worth, TX 76107, USA; irinkoby@gmail.com (I.T.); asama6@gmail.com (A.T.)

**Keywords:** in situ self-assembly, nanoparticles, lopinavir, ritonavir, subcutaneous, injection, HIV

## Abstract

Most antiretroviral medications for human immunodeficiency virus treatment and prevention require high levels of patient adherence, such that medications need to be administered daily without missing doses. Here, a long-acting subcutaneous injection of lopinavir (LPV) in combination with ritonavir (RTV) using in situ self-assembly nanoparticles (ISNPs) was developed to potentially overcome adherence barriers. The ISNP approach can improve the pharmacokinetic profiles of the drugs. The ISNPs were characterized in terms of particle size, drug entrapment efficiency, drug loading, in vitro release study, and in vivo pharmacokinetic study. LPV/RTV ISNPs were 167.8 nm in size, with a polydispersity index of less than 0.35. The entrapment efficiency was over 98% for both LPV and RTV, with drug loadings of 25% LPV and 6.3% RTV. A slow release rate of LPV was observed at about 20% on day 5, followed by a sustained release beyond 14 days. RTV released faster than LPV in the first 5 days and slower than LPV thereafter. LPV trough concentration remained above 160 ng/mL and RTV trough concentration was above 50 ng/mL after 6 days with one subcutaneous injection. Overall, the ISNP-based LPV/RTV injection showed sustained release profiles in both in vitro and in vivo studies.

## 1. Introduction

Human immunodeficiency virus (HIV) is a major global public health issue, with approximately 34.7 million people living with it, 690,000 people having died from related illnesses, and 1.7 million people being newly infected worldwide in 2019, according to the World Health Organization [1]. There is currently no cure for HIV infection. However, it can be treated and prevented by using oral HIV medications called antiretroviral therapy (ART). Even though medications can improve the survival of patients infected with HIV, there are adherence issues, such as forgetting to take HIV medications, dosing complexity, side effects, and pill burden [2,3,4], and at least 95% adherence is necessary for effective HIV viral suppression [5].

Lopinavir (LPV) is one of the potent protease inhibitors (PIs) used in the treatment of HIV infection. LPV has very poor bioavailability when it is administered orally due to high first-pass metabolism, primarily mediated by the cytochrome 450 3A4 (CYP3A4). To solve this bioavailability problem, ritonavir (RTV) is co-administered with LPV, which enhances the transport of LPV from the intestinal lumen to the systemic circulation by inhibiting the CYP3A4-mediated metabolism of LPV [6]. RTV increases LPV drug exposure up to 77-fold in both antiretroviral-naïve and experienced subjects [7]. Both LPV and RTV are classified as class II drugs; thus, they are poorly water soluble and have high permeability. Kaletra^®^ is a once-a-day, fixed-dose combination of LPV/RTV at a 4:1 ratio (LPV/RTV, *w/w*), commercially available in either a tablet or an oral solution form. However, the use of oral LPV/RTV medications does not address the issue of adherence to ART. Moreover, the potential side effects for LPV/RTV are gastrointestinal (GI) disturbances, such as nausea, vomiting, and diarrhea [8].

**Figure 1:**
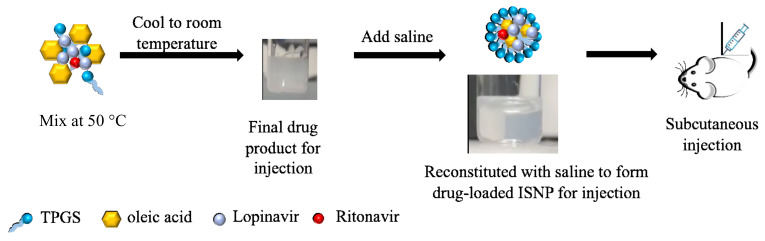
Illustration of manufacturing the ISNP-based LPV/RTV injection and the formation of LPV/RTV-loaded ISNPs.

Long-acting injectable nanoformulations for ART offer alternative therapeutic options for the treatment of HIV. Injectable nanoparticles have the potential to improve the pharmacokinetic properties of drug molecules and overcome GI side effects [9,10], thereby improving drug adherence. Furthermore, with an injection route, long-acting injections will avoid first-pass metabolism when compared to oral drugs [11]. We developed oral solid granules of the combination of LPV and RTV. Once the solid granules came into contact with water, they produced drug-loaded in situ self-assembly nanoparticles (ISNP). The ISNPs were capable of encapsulating both LPV and RTV with over 95% entrapment efficiency (EE) and were physically stable over 6 months at room temperature [12]. The LPV/RTV granules increased the bioavailability of LPV over 2.5-fold compared with a commercial Kaletra^®^ tablet. The excipients, including D-α-tocopherol polyethylene glycol 1000 succinate (TPGS) and oleic acid, which are approved by the Food and Drug Administration (FDA) for oral administration, were chosen as the surfactant and lipid to make the granules. Aeroperl 300 as an absorption carrier was added into an intermediate mixture of oleic acid (lipid), TPGS (surfactant), and LPV/RTV to prepare LPV/RTV solid granules. Interestingly, the intermediate mixture did not contain nanoparticles, but upon reconstitution with saline, it also produced drug-loaded ISNPs. Therefore, it is possible to use the intermediate mixture to prepare an ISNP-based LPV/RTV product for subcutaneous injection (Figure 1). Because water is removed from formulation preparation, the final drug product for injection does not contain nanoparticles. Thus, the long-term stability of nanoparticles during storage will not be an issue for this ISNP approach. In addition, LPV/RTV ISNP granules were stable over 6 months, demonstrating that LPV and RTV were compatible with the excipients used in the granules. When using the same lipid and surfactant as in the granules, the ISNP-based LPV/RTV injection will be stable over 6 months.

The purpose of this study was to develop a novel long-acting injectable nanoformulation, ISNP-based LPV/RTV injection to encapsulate LPV and RTV by using ISNP nanotechnology. The ISNP-based LPV/RTV injection was characterized in terms of particle size, entrapment efficiency (EE), drug loading (DL), in vitro release study, and in vivo pharmacokinetic study. 

## 2. Materials and Methods

### 2.1. Materials

TPGS was given as a gift from BASF (Ludwigshafen, Germany). Oleic acid, PBS, acetonitrile, and methanol (MeOH) were purchased from Fisher Scientific (Fair Lawn, NJ, USA). LPV and RTV were purchased from AK Scientific (Union City, CA, USA) and USP (Rockville, MD, USA), respectively. The Amicon Ultra-0.5 centrifugal filter unit with a molecular weight cutoff of 100 kDa was purchased from Millipore (Bedford, MA, USA). KH_2_PO_4_ and NaOH were purchased from Sigma-Aldrich (St. Louis, MO, USA).

### 2.2. Preparation of ISNP-Based LPV/RTV Injection

The injection was prepared using an ISNP method as previously reported with modification under a sterile hood [12]. Briefly, 50 mg of oleic acid, 100 mg of TPGS, 50 mg of LPV, and 12.5 mg of RTV (LPV:RTV = 4:1, *w/w*) were weighed and mixed in a sterile glass vial at 50 °C for 10 min and cooled to room temperature to form an ISNP-based LPV/RTV injection that can be stored at room temperature as an injection drug product.

### 2.3. Characterization of LPV/RTV ISNPs

To determine particle size and size distribution, 1 mL of 0.9% NaCl was added into the vial containing the ISNP-based LPV/RTV injection and vortexed to obtain LPV/RTV ISNPs. Then, 100 μL of the LPV/RTV ISNPs were diluted with 1 mL of Milli-Q water and measured by using a Delsa Nano C Particle Analyzer (Beckman Coulter Inc., Brea, CA, USA) at 165° light scattering at 25 °C.

To further confirm the formation of drug ISNPs, transmission electron microscopy (TEM) was used to measure the particle. A carbon/formvar-coated copper grid was covered by LPV/RTV ISNPs and then negatively stained. The grid was visualized with a Tecnai G^2^ spirit TEM (ThermoFisher, Hillsboro, OR, USA) equipped with a LaB_6_ source at 120 kV using a Gatan ultrascan CCD camera.

### 2.4. Determination of Entrapment Efficiency and Drug Loading

To measure drug EE in the LPV/RTV ISNPs, free LPV and RTV were separated from the LPV/RTV ISNPs using a membrane filtration device (Amicon Ultra-0.5 centrifugal filter unit with molecular weight cutoff 100 kDa) by centrifugation at 14,000 rpm for 5 min at 4 °C as previously reported [12]. After centrifugation, the LPV/RTV ISNPs (about 20 µL) in the membrane were destroyed by adding 400 µL of MeOH to measure the entrapped drugs by a validated HPLC method as previously reported [12]. EE was calculated as follows:

$$\mathrm{EE}\%=\frac{\mathrm{drug}\;\mathrm{entrapped}\;\mathrm{in}\;\mathrm{ISNPs}}{\mathrm{total}\;\mathrm{drug}\;\mathrm{in}\;\mathrm{ISNPs}}\times 100\%\;\left(w|w\right)$$

To determine DL, 1 part of the ISNP-based LPV/RTV injection was dissolved in 33.3 parts of methanol by aggressively vortexing for 5 min for HPLC measurement. DL was calculated as follows:

$$\mathrm{DL}\%=\frac{\mathrm{measured}\;\mathrm{drug}\;\mathrm{weight}}{\mathrm{total}\;\mathrm{weight}\;\mathrm{of}\;\left(\mathrm{drug}+\mathrm{excipients}\right)}\times 100\%\;\left(w|w\right)$$

### 2.5. In Vitro Release Studies

LPV/RTV release studies (*n* = 4) were completed at 37 °C using PBS buffer as the release medium. Two hundred microliters of the ISNP-based LPV/RTV injection were added into 20 mL PBS and shaken at 135 rpm at 37 °C. Five hundred microliters of sample were withdrawn at predetermined time intervals and added into an Amicon Ultra-0.5 centrifugal filter unit to separate free drug from drug-loaded ISNPs. The entrapped drug in the LPV/RTV ISNPs was measured as described above. The released drug was calculated as follows:

The released drug = 100% − entrapped drug%

### 2.6. Pharmacokinetic Studies

Animal studies were performed in compliance with guidelines set by the University of North Texas Health Science Center Institutional Animal Care and Use Committees (2013/14-12-A05 approved on 2 April 2014). Rats were housed in groups of two under a 12 h light/dark cycle with free access to food and water for one week before use. Sprague–Dawley rats (males, 276–300 g, *n* = 4) were purchased from Charles River Laboratories (Wilmington, MA, USA). Pharmacokinetic studies were conducted to evaluate the pharmacokinetic profiles of LPV and RTV from the ISNP-based LPV/RTV injection. Rats were treated with ~100 µL of the ISNP-based LPV/RTV injection to provide 100 mg/kg of LPV by subcutaneous injection. After the injection, blood was collected at 0, 1, 2, 3, 5, 6, and 24 h and 2, 3, 4, 5, and 6 days. The blood was immediately centrifuged at 3400 rpm for 5 min at 4 °C, and 100 µL of plasma samples were transferred to a new tube and stored at −20 °C until further analysis within 3 weeks. LPV and RTV concentrations in the plasma were determined by liquid chromatography-mass spectrometry (LC-MS, an Agilent G6460 Triple Quad LC-MS system) (Agilent Technologies, Santa Clara, CA, USA) using a previously reported method [12].

### 2.7. Pharmacokinetic Data Analysis

Pharmacokinetic parameters (*C_max_, T_max_*, *T*_1/2_, and *AUC*_0-∞_) of LPV and RTV were analyzed using noncompartmental analysis methods using Phoenix WinNonlin version 8.0 (Certara USA, Inc., Princeton, NJ, USA).

### 2.8. Statistical Analysis

The results are presented as mean ± standard deviation (SD). The data were analyzed by using a two-tailed *t*-test at 95% confidence level. *p* < 0.05 was considered a statistically significant difference.

## 3. Results

### 3.1. Characterization of LPV/RTV ISNPs

**Figure 2:**
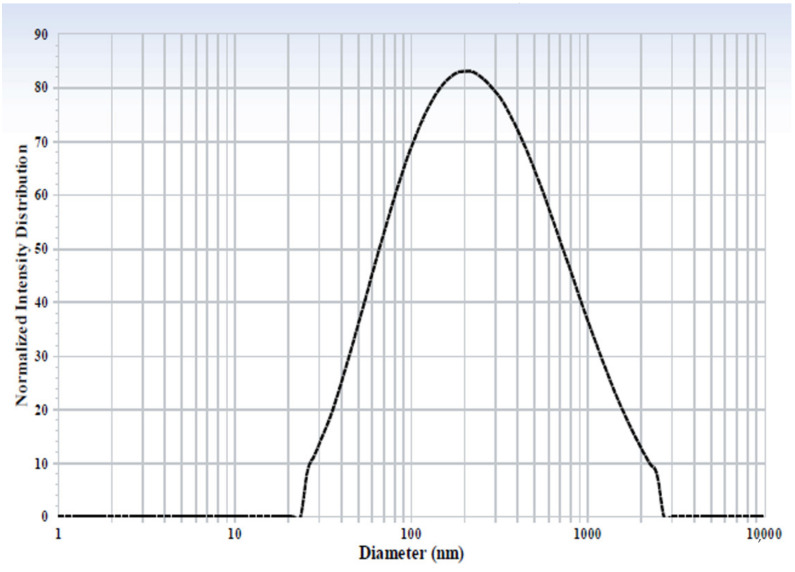
Particle size and size distribution of LPV/RTV ISNPs.

**Figure 3:**
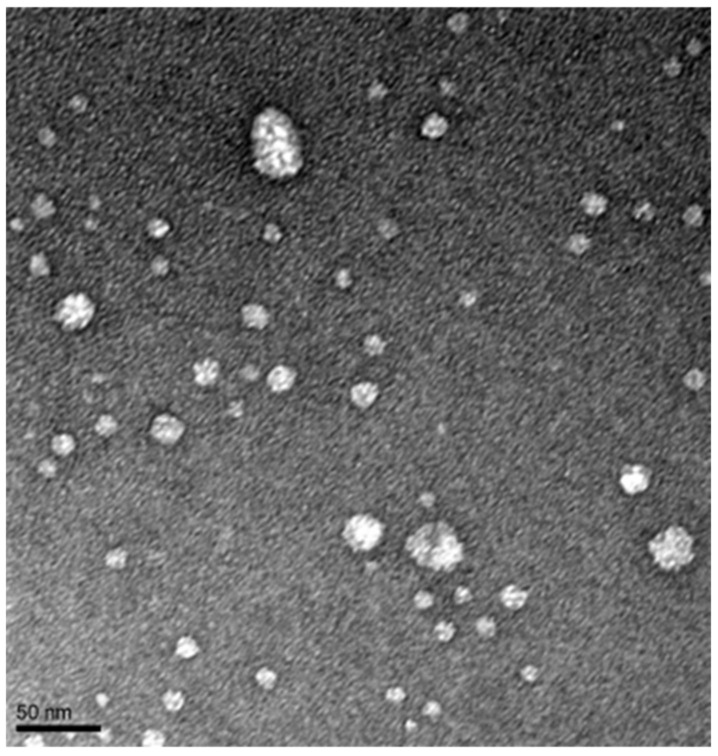
TEM image of LPV/RTV-loaded ISNPs.

As shown in Table 1, particle size of LPV/RTV ISNPs was 167.8 ± 9.9 nm (*n* = 3) after the ISNP-based LPV/RTV injection was reconstituted with 0.9% NaCl solution. LPV/RTV ISNPs were monodispersed with a narrow size distribution indicated by a polydispersity index (P.I.) of less than 0.35 (Figure 2). Particle sizes were consistent with the result from LPV/RTV ISNP granules in our previous study [12]. As shown in Table 1, 98.9% LPV and 98.7% RTV were entrapped in LPV/RTV ISNPs. The measured DLs were 23.5% LPV and 5.9% RTV. In addition, the formation of spherical ISNPs was confirmed by TEM imaging (Figure 3).

### 3.2. In Vitro Release Studies

**Figure 4:**
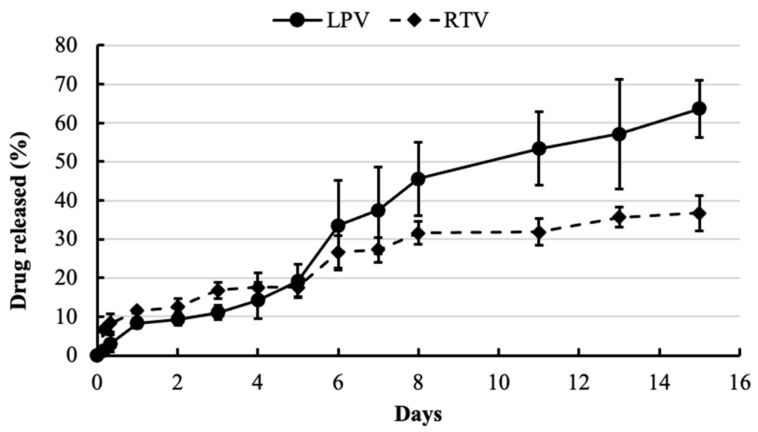
Release of LPV and RTV from the ISNP-based LPV-RTV injection in a simulated physiological condition. The study was performed in PBS (pH 7.4) at 37 °C for 15 days. Released LPV and RTV were measured by HPLC. Data are presented as the mean ± SD (*n* = 4).

The drug release from the injection was tested in PBS buffer in a physiological condition (pH 7.4). Once the ISNP-based LPV/RTV injection came into contact with the release medium, it generated LPV/RTV ISNPs that controlled the drug release. The cumulative LPV and RTV release from the injection is shown in Figure 4. LPV and RTV exhibited sustained release profiles. A slow release rate of LPV was observed at about 20% on day 5 for LPV, followed by a sustained release beyond 14 days. RTV released faster than LPV in the first 5 days but slower than LPV thereafter. According to our previous studies [12], the solubility of LPV in the excipients of the ISNPs was higher than that of RTV. Thus, once in contact with the release medium, RTV showed a faster release in the first 5 days. However, in the injection, the DLs of LPV and RTV were 25% and 6.25%, respectively. Thus, the ISNPs could have a stronger interaction with RTV at this relatively low DL compared to LPV, which could lead to slower release of RTV after the first 5 days. The injection did not show any burst release patterns, confirming that drugs were homogeneously dispersed with excipients. This also proved that LPV and RTV were loaded inside the ISNPs and that the drugs were not on the surface of the ISNPs.

### 3.3. Pharmacokinetics

**Figure 5:**
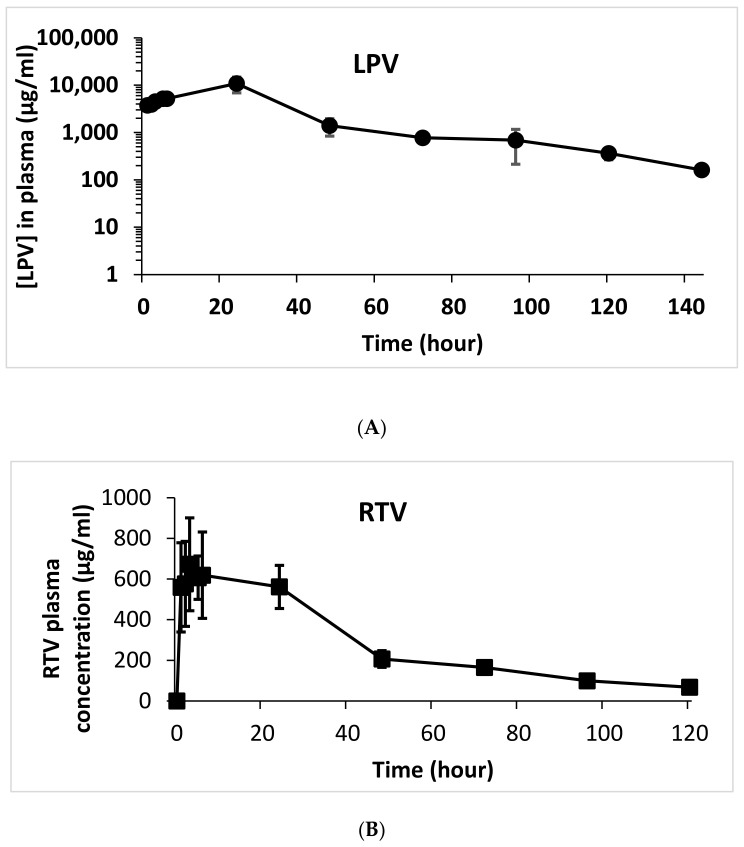
Pharmacokinetics of the ISNP-based LPV/RTV injection in rats over 6 days for LPV (**A**) and RTV (**B**). The ISNP-based LPV/RTV injection was given to rats by subcutaneous injection at 100 mg/kg of LPV. Plasma samples were analyzed by a validated LC-MS method. Data are presented as the mean ± SD (*n* = 4).

There are currently no LPV/RTV long-acting injections. Thus, we do not have a standard formulation for comparison as well as an injection dose as a reference. The oral dose of LPV in rats is 10 mg/kg [12]. Thus, a dose (100 mg/kg) that was 10-fold higher than the oral dose was selected to test the ISNP-based LPV/RTV injection to provide enough drug concentrations over one week. The efficacy of antiretroviral drugs is determined by *C_trough_*, which is the lowest drug concentration during the course of a dosing interval, typically occurring immediately prior to the next scheduled dose. Thus, we used *C_trough_* as the criterion to evaluate the ISNP-based LPV/RTV injection. Plasma concentrations of LPV and RTV are plotted in Figure 5A,B. After 6 days, the *C_trough_* of LPV remained above 160 ng/mL, and the C_trough_ of RTV was about 50 ng/mL with one subcutaneous injection. The pharmacokinetic parameters of noncompartmental analysis are listed in Table 2. RTV (*T*_1/2_ = 44.9 h) showed slower reduction of blood concentration compared to that of LPV (*T*_1/2_ = 23 h), which was correlated to the observation in the in vitro release profile (Figure 4). In the injection sites, no irritation or inflammation was observed. Rats did not show any sign of illness, dehydration, hypothermia, pain, or distress during the treatments.

## 4. Discussion

Lipid nanoparticles ideal for poorly water-soluble drugs. Several methods have been used to prepare lipid nanoparticles. However, these nanoparticles are traditionally prepared in a liquid form, and consequently long-term storage in solution and stability of nanoparticle sizes are problematic. To solve these issues, techniques such as freeze drying and spray drying [13,14] have been used to dry nanoparticles. The disadvantages of these approaches are that they are time consuming, and there is a size increase after reconstituting the dried nanoparticles. ISNP technology offers a novel procedure to prepare nanoparticles without using water. In this nanotechnology, a mixture of oleic acid and TPGS spontaneously forms ISNPs with appropriate particle size and distribution when in contact with liquid (e.g., body fluid). Therefore, ISNP technology overcomes the aforementioned issues with traditional lipid nanoparticles. For example, the stability of particle size during storage is of less concern for the ISNP-based injection, because the mixture of drugs with lipid and surfactant is the final formulation that is kept on the shelf, and the ISNPs form after injection. The preparation procedure is very simple. The excipients including oleic acid and TPGS are natural materials. Thus, novel ISNP technology has a great potential to produce parenteral nanoformulations for clinical translation.

Development of fixed-dose combination nanoformulations for injection is quite challenging. Drugs in a fixed-dose combination have to be encapsulated into one nanoparticle. Very often, the nanoparticle may be suitable for one drug, but not for the other drug, because drugs have different physicochemical properties. LPV and RTV have been encapsulated into PLGA nanoparticles; however, less than 46% of LPV and RTV were entrapped into the nanoparticles [15]. Although RTV is used to improve oral absorption of LPV, its mechanism is based on the inhibition of CYP3A4. For subcutaneous injection, the first-pass effect caused by CYP3A4 degradation may not be pronounced, but for long-acting injections, drugs will circulate in the body at least over one week and will interact with CYP3A4 during the circulation time. Thus, RTV could still be needed to reduce the enzyme degradation of LPV over time. To test the feasibility of the ISNPs for long-acting injections encapsulating both LPV and RTV, the ratio of 4/1 (LPV/RTV, *w/w*) that is used for oral combination was kept in this study. With the ISNP technology, drugs are dissolved in the excipients. When the ISNP-based injection comes into contact with body fluid after injection, oleic acid and TPGS release and spontaneously form the ISNPs through a self-assembly process and, during this process, drugs are entrapped into the ISNPs. This novel mechanism leads to the minimum amounts of excipients with higher EE% and DL%. In this study, we successfully entrapped over 98% of both LPV and RTV into one nanoparticle with 25% of DL for LPV and 6.3% of DL for RTV in the injection. Both oleic acid and TPGS are common excipients for pharmaceutical development. The manufacturing process of LPV/RTV injection (Figure 1) is simple and scalable using common pharmaceutical equipment. As the final drug injection does not contain nanoparticles, particle stability is not an issue, and, thus, the final injection does not need to be lyophilized, unlike liposomal drug formulations. Thus, the cost of manufacturing and storage of the ISNP-based LPV/RTV injection would be low. The new ISNPs could be a potential drug delivery system to produce fixed-dose combinations for injections to treat HIV.

In addition to the aforementioned advantages, we demonstrated the sustain release properties of the ISNP-based LPV/RTV injection in this study. According to the in vitro release, about 60% LPV and 40% RTV were released from the injection after 14 days. The further rat pharmacokinetic study confirmed that the injection maintained the drug concentrations over 6 days by one subcutaneous injection. Because of the limited fluid volume in the injection site, the injection could form a “depot” and slowly release the drugs, oleic acid, and TPGS over time. The half-life of LPV and RTV was longer compared to that of previous LPV/RTV oral granules [12]. This indicated that the overall rate of elimination of the ISNP-based LPV/RTV injectable formulation was slow. Long-acting injectable formulations offer alternative therapeutic options for the treatment of HIV. Nanoparticulate drugs administered through subcutaneous injection can be retained in the lymphatic system and improve intracellular exposure of the drugs, which could overcome HIV lymphatic drug insufficiency as with oral dosing [16,17]. Additionally, LPV and RTV are both lipophilic molecules that move easily in lipoid compartments in underlying subcutaneous tissue [18]; thus, the ISNP-based LPV/RTV injection will facilitate drug movement through the lymphatic circulation. We did not observe any redness, swelling, or irritation on the rat skin, suggesting that the injection did not cause any skin hypersensitivity reaction or trigger an immune response. The weekly dosed ISNP-based LPV/RTV injection could improve medication adherence, reduce side effects, lower dose medication on the next dosing schedule, and help in the management of HIV.

## 5. Conclusions

In this study, an ISNP-based LPV/RTV injection was successfully prepared for the purpose of a long-acting injection by using novel ISNP nanotechnology. After mixing saline with the ISNP-based LPV/RTV injection, LPV/RTV ISNPs formed with a particle size of 168 nm and over 98% of EE% for both drugs. The preparation of the ISNPs is simple and scalable with few concerns on nanoparticle stability. The LPV/RTV ISNPs exhibited sustained release behavior in an in vitro physiological condition as well as after subcutaneous injection into rats. Therefore, novel ISNP nanotechnology has great potential to produce injectable nanoformulations for long-acting fixed-dose combinations. LPV/RTV ISNPs could be a new nanoformulation to improve patient adherence and therapeutic effectiveness.

## Figures and Tables

**Table 1:** Physicochemical properties of LPV/RTV ISNPs. Data are presented as the mean ± SD (*n* = 3).

Drugs	Theoretical DL%	Particle Size (nm)	P.I.	EE%	Measured DL%
LPV	25	167.8 ± 9.9	0.310 ± 0.024	98.9 ± 0.5	23.5
RTV	6.3			98.7 ± 0.2	5.9

**Table 2:** Pharmacokinetic parameters of LPV and RTV following subcutaneous injection of the ISNP-based LPV/RTV injection at a dose of 100 mg/kg LPV in rats.

Drugs	*C_max_* (µg/mL)	*AUC*_0-∞_ (µg·h/mL)	*T_max_* (h)	*T*_1/2_ (h)
**LPV**	10.9	384.7	24	23.0
**RTV**	0.7	37.2	3	44.9

## Data Availability

Not applicable.
